# Dietary factors associated with inflammatory laryngeal disease in South Korea

**DOI:** 10.1371/journal.pone.0244216

**Published:** 2020-12-31

**Authors:** Soo Yeon Jung, Min-ho Kim, So Jeong Lee, Eun Hee Ha, Han Su Kim

**Affiliations:** 1 Department of Otorhinolaryngology and Head and Neck Surgery, College of Medicine, Ewha Womans University, Seoul, South Korea; 2 Ewha Medical Research Institute, Ewha Womans University, Seoul, South Korea; 3 Department of Occupational and Environmental Medicine, College of Medicine, Ewha Womans University, Seoul, South Korea; San Raffaele Roma Open University, ITALY

## Abstract

Laryngeal inflammation causes not only benign diseases of the larynx, such as laryngitis and granuloma, but also malignancy. Dietary factors are known to control or modulate the inflammatory reaction in the body. To date, the association between laryngeal inflammation and dietary factors has not been reported using nationwide population-based data. The aim of this study was to analyze the association between several dietary factors and inflammatory laryngeal disease in the Korean population. This study analyzed the data from Korean National Health and Nutrition Examination Surveys which is cross-sectional nationwide-population-based study. Association between the dietary nutrient intake and the prevalence of inflammatory laryngeal diseases was analyzed in 21,116 participants who underwent a laryngoscopy and filled in the dietary intake questionnaires. Of the 21,116 participants included in the analysis, 758 (3.59%) were diagnosed with inflammatory laryngeal disease. Prevalence of inflammatory laryngeal disease was higher in men (4.58%) than in women (2.84%). The mean age of patients was 53.77 years. When analyzing the risk using propensity score matching, ILD group tend to consume more coffee and to intake less fiber and iron than normal group. On Logistic regression analysis, an increased intake of carbohydrate, fiber, and iron was associated with lowered risk of having ILD in female. The association between inflammatory laryngeal disease and dietary factors was prominent in the group aged ≥50 years and female. Increased intake of fiber, iron, and vitamin A were associated with lower risk in the group aged ≥50 years. In female, increased intake of fiber, iron were associated with lower risk of having ILD. In the group aged ≤50 years, only an increased consumption of makgeolli, Korean traditional rice wine, was associated with a higher risk of ILD.

## Introduction

Laryngeal inflammation causes various symptoms, from mild throat discomfort to severe choking sensation, for which patients visit a laryngologist [1, 2]. There are numerous causes of inflammation of the larynx: viral or bacterial infection, allergy, acid or bile reflux, mechanical irritation, chemical irritants such as smoking or air pollution, and systemic disease [1, 3–6]. Inflammation of the larynx can develop into chronic laryngitis or contact granuloma. Chronic laryngitis is a pathological inflammatory change of the mucosa without disruption of the mucosal lining [7, 8]. Contact granuloma develops following inflammation after initial trauma to the mucosa at the vocal process [9, 10]. The prevalence of chronic laryngitis and contact granuloma has been found to be 3.37–3.51% and 0.11%, respectively [1, 5, 11].

These chronic inflammatory diseases are difficult to manage. Proton pump inhibitors, H2 blockers, and prokinetics have been used to prevent inflammatory damage from acid or pepsin reflux. Mucolytic agents, anti-allergic agents, and anti-inflammatory medication such as corticosteroids or nonsteroidal anti-inflammatory drugs have also been prescribed to reduce chronic laryngeal inflammation. Among these agents, corticosteroids demonstrated treatment effect; however, long-term use of corticosteroids can lead to multiple systemic complications [8, 12].

Dietary factors are known to modulate the immune reaction. Many studies have reported the anti- or pro-inflammatory effects of dietary factors. Therefore, changes in dietary intake could reduce the reliance on anti-inflammatory drugs without systemic complications or risk of long-term anti-inflammatory medication use. With regard to dietary nutrient intake and inflammation, the association between alcohol and laryngeal disease is well known [13, 14]. However, no association between laryngeal inflammation and different beverage types (carbonated soft drinks, tea, coffee, makgeolli [Korean traditional rice wine], beer, or soju [Korean whisky]) has been reported. The influence of dietary nutrient intake on inflammatory bowel disease [15], systemic lupus erythematous [16], arthritis, and even sepsis [17, 18] has been reported. However, for laryngeal disease, only an association between chronic laryngitis and vitamin A intake has been identified, with the association between laryngeal inflammation and other nutrients remaining to be clarified [19]. Therefore, our aim in this study was to investigate the prevalence of inflammatory laryngeal disease (ILD) and dietary factors including beverage type, water, calories, and micronutrients, associated with ILD, and its prevention, using data from the Korea National Health and Nutrition Examination Survey (KNHANES), which includes interview data about dietary consumption and laryngoscopic examination from 2008 to 2012.

## Materials and methods

### Data source and study population

Data were obtained from the fourth and fifth deployment of the KNHANES, conducted by the Korea Centers for Disease Control and Prevention, between 2008 and 2012. KNHANES is a household-based survey conducted by a field survey team consisting of a nurse examiner, an ophthalmologist, and an otolaryngologist in a mobile examining van. The survey includes a health interview, a nutritional survey, a physical examination, blood sampling, pulmonary function test, dental examination, otolaryngological survey, and ophthalmological examination. The details of this survey have been described in a previous study [20]. In our study, only individuals over the age of 19 years were included, as laryngoscopic examination was not performed in children. As baseline characteristics, we collected the data for sex, age, educational level (graduation from elementary, middle, or high school or college), area of residence (state and city), household income level (classified into quartiles Q1, Q2, Q3, and Q4, with Q1 being the lowest income quartile and Q4 being the highest income quartile), body mass index (BMI), and comorbidities (hypertension and diabetes mellitus). Our study was approved by the institutional review board of the Korea Centers for Disease Control and Prevention, and all participants provided written informed consent.

### Definition of the ILD using laryngoscopic examination

Laryngoscopic examination were performed using a 4-mm, 70-degree-angled rigid endoscope, with a charge-coupled device (CCD) camera. Trained otorhinolaryngology senior residents conducted the laryngoscopic evaluation while recording it. The findings were diagnosed as 12 categories (vocal nodule, polyp, cyst, palsy, Reinke’s edema, sulcus vocalis, laryngeal papilloma, laryngitis, epiglottic cyst, contact granuloma, hyperkeratosis (leukoplakia), and laryngeal cancer) according to the guideline of the Epidemiological Survey Committee of the Korean Society of Otorhinolaryngology-Head and Neck Surgery. Chronic laryngitis and contact granuloma were included in the definition of ILD. Chronic laryngitis was defined as chronic laryngeal inflammation with erythema, edema, or thick mucus findings on laryngoscopic examination [5, 11, 21]. Contact granuloma was defined as polypoid mass on the posterior part of the true vocal fold, especially on the vocal process. Recorded laryngoscopic images were sent to the Epidemiological Survey Committee of the Korean society of Otorhinolaryngology-Head and Neck Surgery, and two expert laryngologists verified the laryngoscopic images on a second check.

### Dietary data using 24-hour dietary recall and food frequency questionnaire

The KNHANES collected dietary data using the 24-h recall (participants recalled their dietary intake over the 24 hours prior to the visit) and the food frequency questionnaire (FFQ) (participants reported their food consuming patterns including food category, frequency, and amount). Using the 24-h recall data, daily dietary intake of nutrients (calories, protein, fat, carbohydrates, fiber, calcium, phosphorus, iron, potassium, vitamin A, carotene, retinol, thiamin, riboflavin, niacin, and vitamin C) were estimated by KNHANES database using the CAN-Pro 3.0 Nutrient Database (The Korean Nutrition Society, Seoul, South Korea), which is similar to Mosby's NutriTrac Nutrition Analysis Software (Mosby, 2005, St. Louis, MO, USA). From the FFQ, among 63 food items, data about consumption of carbonated soft drink, coffee, tea, beer, soju, and makgeolli consumption was collected for inclusion in our analysis. The daily, weekly, monthly, and yearly intake frequency was checked. The raw questionnaire is available at https://knhanes.cdc.go.kr/.

### Statistical analysis

SAS (version 9.4, SAS Institute Inc. Cary, NC, USA) was used to perform all statistical analyses. Descriptive statistics were used to assess the baseline characteristics of the study population. The intake amount of beverages and nutrients, smoking (pack-years), and the Alcohol Use Disorders Identification Test (AUDIT) scores were analyzed as continuous variables, and expressed as the mean and standard deviation.

Sex, income level, education, BMI, diabetes, and hypertension were analyzed as categorical variables. Continuous variables and ordinary variables were expressed using mean and standard deviation and categorical variables were expressed using count and percentage. The propensity scores obtained from the fitted model were used to match the respondents in the ILD group and non-ILD groups until achieving accepted balances using logistic regression model for all covariates (demographic characteristics of the respondents). The nearest neighbor 1:4 matching with caliper size of 0.25 and without replacement. Student’s t-test and chi-squared test were used to compare baseline characteristics. Multiple logistic regression models were used to calculate odds ratio (OR), 95% confidence interval (CI), and corresponding p-value, controlling for age and sex as covariates.

## Results

### Baseline characteristics of inflammatory laryngeal disease in the study participant

Among the KNHANES 2008–2012 participants, 21,116 were over 19 years old and underwent laryngoscopy. Of these, 9105 (43%) were male and 12,011 (57%) were female. On laryngoscopic evaluation, chronic laryngitis was diagnosed in 740 and contact granuloma in 20, with both conditions diagnosed simultaneously in two patients. Men had higher prevalence of ILD (4.58%) than women (2.84%). The mean age of the ILD patients was higher than that of the controls (53.77 ± 14.81 and 49.18 ± 16.54 years, p <0.001). Higher BMI and AUDIT score, and lower educational level, were significantly associated with ILD. ILD patients had higher blood pressure and blood sugar than the participants in the non-ILD group (Table 1).

**Table 1:** Characteristics of participants with and without ILD.

		with Inflammatory laryngeal disease (n = 758, 3.59%)		without inflammatory laryngeal disease (n = 20358, 96.41%)		*P* value
Health status and demographic characteristics		number	% of total	number	% of total	
**Sex**						
Male		417	55.01	8688	42.68	< .001
Female		341	44.99	11670	57.32	
**Age (mean ± SD)**		53.77 ± 14.81		49.18 ± 16.54		
	≥50	478	63.06	9776	48.02	< .001
	<50	280	36.94	10582	51.98	
**Laryngeal disease**						
	Laryngitis	740	97.63	0		
	Contact granuloma	20	2.64	0		
**Educational level**						
	Elementary school	244	32.19	5310	26.08	< .001
	Middle school	97	12.80	2204	10.83	
	High school	248	32.72	6910	33.94	
	College	156	20.58	5677	27.89	
**Income level**						
	Q1	195	25.73	4933	24.23	.089
	Q2	212	27.97	5059	24.85	
	Q3	167	22.03	5089	25.00	
	Q4	177	23.35	5002	24.57	
**Residence**						
	Rural	414	54.62	11329	55.65	.575
	Urban	344	45.38	9029	44.35	
**Body mass index (mean ± SD)**		24.18 ± 3.39		23.55 ± 3.36		
	<18.5	27	3.56	994	4.88	< .001
	18.5–25	439	57.92	13060	64.15	
	25–30	253	33.38	5505	27.04	
	30≤	37	4.88	745	3.66	
**Alcohol, AUDIT (mean ± SD)**		5.80 ± 7.28		5.45 ± 6.69		.195
**Smoking, packyear (mean ± SD)**		12.70 ± 20.14		7.63 ± 15.00		< .001
**Hypertension**						
	Normal	261	34.43	8955	43.99	< .001
	Prehypertension	201	26.52	4900	24.07	
	Hypertension	287	37.86	6258	30.74	
**Diabetes**						
	Normal	449	59.23	13597	66.79	< .001
	Prediabetes	154	20.32	3418	16.79	
	Diabetes	104	13.72	1840	9.04	

Q1, lowest quartile.

Q4, highest quartile.

AUDIT, Alcohol Use Disorders Identification Test.

Among the 758 patients, 626 were successfully matched to 2504 participants without ILD. After matching, there was no statistical differences in demographics and baseline characteristics between the two groups (Table 2).

**Table 2:** Comparison of characteristics of participants with and without ILD after matching.

		with Inflammatory laryngeal disease (n = 626)		without inflammatory laryngeal disease (n = 2504)		*P* value
Health Status and demographic characteristics		number	% of total	number	% of total	
**Sex**						
Male		335	53.51	1265	50.52	.180
Female		291	46.49	1239	49.48	
**Age (mean ± SD)**		54.53±14.48		54.52±15.64		.991
	≥50	216	34.50	858	34.27	.910
	<50	410	65.50	1646	65.73	
**Laryngeal disease**						
	Laryngitis	611	97.60	0		
	Contact granuloma	15	2..40	0		
**Educational level**						
	Elementary school	210	33.98	901	36.24	.757
	Middle school	88	14.24	337	13.56	
	High school	195	31.55	751	30.21	
	College	125	20.23	497	19.99	
**Income level**						
	Q1	154	24.76	578	23.33	.864
	Q2	175	28.14	691	27.89	
	Q3	135	21.70	555	22.40	
	Q4	158	25.40	654	26.39	
**Residence**						
	Rural	340 (54.31)	54.62	1386 (55.35)	55.65	.640
	Urban	286 (45.69)	45.38	1118 (44.65)	44.35	
**Body mass index (mean ± SD)**		24.17±3.39		24.14±3.35		.823
	<18.5	20	3.19	80	3.20	.958
	18.5–25	357	57.03	1439	57.63	
	25–30	219	34.98	849	34.00	
	30≤	30	4.79	129	5.17	
**Alcohol, AUDIT (mean ± SD)**		5.53±7.20		5.29±6.62		.449
**Smoking, packyear (mean ± SD)**		12.76±20.10		11.43±19.37		.129
**Hypertension**						
	Normal	208	33.66	860	34.57	.895
	Prehypertension	169	27.35	680	27.33	
	Hypertension	241	39.00	948	38.10	
**Diabetes**						
	Normal	372	63.05	1522	64.14	.774
	Prediabetes	131	22.20	495	20.86	
	Diabetes	87	14.75	356	15.00	

Q1, lowest quartile.

Q4, highest quartile.

AUDIT, Alcohol Use Disorders Identification Test.

#### Association between dietary factors and ILD

As shown in Table 3, compared to participants in the non-ILD group, participants with ILD tended to consume more coffee than controls and to have a significantly lower iron and fiber intake.

**Table 3:** Beverage and nutrients median consumption pattern of participants.

Beverage and nutrients consumed		Median (SD)		*P* value
		with Inflammatory laryngeal disease (n = 626)	without inflammatory laryngeal disease (n = 2504)	
**Beverage**				
	Carbonated soft drink	0.51±1.39	0.54±1.26	0.637
	Coffee	9.45±7.77	8.77±7.25	0.046*
	Tea	1.87±3.81	1.78±3.73	0.587
	Beer	0.54±1.20	0.55±1.23	0.882
	Soju	1.00±2.02	1.06±2.30	0.556
	Makgeolli	0.34±1.29	0.28±1.20	0.297
**Nutrients**				
	Energy (kcal)	1932.84±822.92	1945.06±816.41	0.738
	Water (g)	934.08±666.15	941.49±645.76	0.798
	Protein (g)	68.08±36.24	68.20±38.61	0.942
	Fat (g)	35.20±29.41	35.42±30.20	0.870
	Carbohydrate (g)	316.26±121.32	323.70±122.43	0.173
	Fiber (g)	7.29±4.49	7.77±5.85	0.027*
	Calcium (mg)	498.09±343.01	495.87±318.62	0.883
	Phosphate (mg)	1156.80±508.25	1158.32±514.03	0.947
	Iron (mg)	14.14±8.69	14.99±11.07	0.041*
	Sodium (mg)	4830.67±3016.27	5009.51±3546.12	0.201
	Potassium (mg)	3043.48±1523.05	3060.65±1615.87	0.810
	Vitamin A (μgRE)	741.98±670.53	796.92±930.44	0.092
	Carotene (μg)	3946.67±3763.15	4165.91±4908.60	0.222
	Retinol (μg)	84.27±141.61	92.40±292.52	0.318
	Thiamin (mg)	1.25±0.74	1.27±0.77	0.533
	Rivoflavin (mg)	1.14±0.70	1.16 ± 0.79	0.724
	Niacin (mg)	16.42±9.17	16.00 ± 9.43	0.375
	Vitamin C (mg)	107.12±105.27	106.28 ± 93.58	0.787

* indicates statistically significant result.

The association between dietary factors and ILD was analyzed using a logistic regression analysis. Decreased fiber and iron intake were associated with an increased risk of ILD. (Table 4) On subgroup analysis by sex, after adjusting for age as a confounder, increased carbohydrate, fiber, and iron intake was associated with lower risk of having ILD among women (OR (95% CI), carbohydrates 0.87 (0.77–0.98); fiber 0.96 (0.94–0.99), and iron 0.85 (0.73–0.99)). Other dietary factors were not significantly associated with an increased or decreased risk of having ILD.

**Table 4:** Dietary factors by nutrients associated with inflammatory laryngeal disease in participants.

		Odds Ratio (95% CI)			
Variables	Unadjusted	Sex group (adjusted for age)		Age group (adjusted for sex)	
		Male	Female	<50y	≥50y
**Beverage**					
Carbonated soft drink	0.97(0.90–1.05)	0.98(0.90–1.07)	0.93(0.79–1.08)	0.97(0.89–1.07)	0.96(0.85–1.10)
Coffee	1.01(1.00–1.02)	1.02(1.00–1.03)	1.01(0.99–1.03)	1.02(1.00–1.04)	1.01(0.99–1.02)
Tea	1.01(0.98–1.03)	0.98(0.95–1.02)	1.03(1.00–1.07)	1.03(0.99–1.06)	0.99(0.96–1.02)
Beer	0.98(0.91–1.06)	0.95(0.87–1.04)	1.07(0.92–1.25)	1.02(0.92–1.14)	0.94(0.84–1.05)
Soju	0.98(0.93–1.02)	0.97(0.93–1.02)	1.04(0.89–1.21)	1.05(0.95–1.16)	0.96(0.91–1.01)
Makgeolli	1.03(0.96–1.10)	1.04(0.97–1.11)	0.93(0.65–1.34)	1.22(1.01–1.48)*	1.01(0.94–1.09)
**Nutrients**					
Energy (kcal)	0.95(0.84–1.07)	1.01(0.87–1.16)	0.83(0.67–1.02)	1.01(0.84–1.20)	0.90(0.77–1.05)
Water (g)	0.96(0.83–1.11)	0.99(0.84–1.17)	0.88(0.68–1.15)	1.06(0.86–1.30)	0.89(0.73–1.08)
Protein (g)	0.99(0.97–1.02)	0.99(0.96–1.02)	1.00(0.95–1.04)	1.00(0.97–1.03)	0.99(0.95–1.02)
Fat (g)	0.99(0.96–1.02)	1.00(0.96–1.04)	0.98(0.92–1.04)	1.00(0.96–1.05)	0.98(0.93–1.02)
Carbohydrate (g)	0.93(0.87–1.01)	0.98(0.89–1.08)	0.87(0.77–0.98)*	0.95(0.84–1.08)	0.92(0.84–1.02)
Fiber (g)	0.98(0.96–1.00)*	1.00(0.97–1.02)	0.96(0.94–0.99)*	0.99(0.96–1.02)	0.98(0.95–1.00)*
Calcium (mg)	1.00(0.97–1.03)	1.01(0.97–1.04)	0.99(0.94–1.03)	1.00(0.95–1.05)	1.00(0.97–1.03)
Phosphate (mg)	0.95(0.79–1.14)	1.00(0.80–1.26)	0.87(0.64–1.18)	1.02(0.77–1.35)	0.91(0.72–1.15)
Iron (mg)	0.91(0.83–1.00)*	0.94(0.84–1.06)	0.85(0.73–0.99)*	0.87(0.74–1.03)	0.93(0.83–1.04)
Sodium (mg)	0.98(0.95–1.01)	0.98(0.94–1.02)	0.98(0.93–1.02)	1.00(0.96–1.04)	0.96(0.92–1.00)*
Potassium (mg)	0.99(0.93–1.04)	1.02(0.95–1.10)	0.94(0.86–1.03)	1.01(0.92–1.10)	0.97(0.91–1.05)
Vitamin A (μgRE)	0.99(0.98–1.00)	0.99(0.98–1.00)	0.99(0.97–1.01)	1.00(0.98–1.02)	0.98(0.97–1.00)*
Carotene (μg)	0.99(0.97–1.01)	0.99(0.96–1.02)	0.98(0.95–1.02)	1.00(0.97–1.03)	0.98(0.95–1.00)
Retinol (μg)	1.00(0.99–1.00)	1.00(0.99–1.00)	1.00(0.99–1.00)	1.00(0.99–1.01)	1.00(0.99–1.00)
Thiamin (mg)	0.94(0.83–1.06)	0.98(0.85–1.14)	0.98(0.85–1.14)	1.03(0.86–1.23)	0.86(0.72–1.02)
Rivoflavin (mg)	1.00(0.88–1.14)	1.03(0.87–1.21)	1.03(0.87–1.21)	1.03(0.83–1.28)	0.98(0.83–1.16)
Niacin (mg)	1.03(0.93–1.14)	1.03(0.91–1.16)	1.03(0.91–1.16)	1.09(0.95–1.26)	1.09(0.95–1.26)
Vitamin C (mg)	1.01(0.92–1.11)	1.07(0.95–1.21)	1.07(0.95–1.21)	0.99(0.85–1.15)	1.03(0.92–1.15)

* indicates statistically significant result.

Subgroup analysis by age (≥50 years and <50 years,) after adjusting for sex as confounders, increased intake of fiber, sodium, and vitamin A were associated with lower risk of having ILD in the ≥50 years group. Among participants <50 years of age, only increased makgeolli intake was associated with increased risk of having ILD.

**Fig 1:**
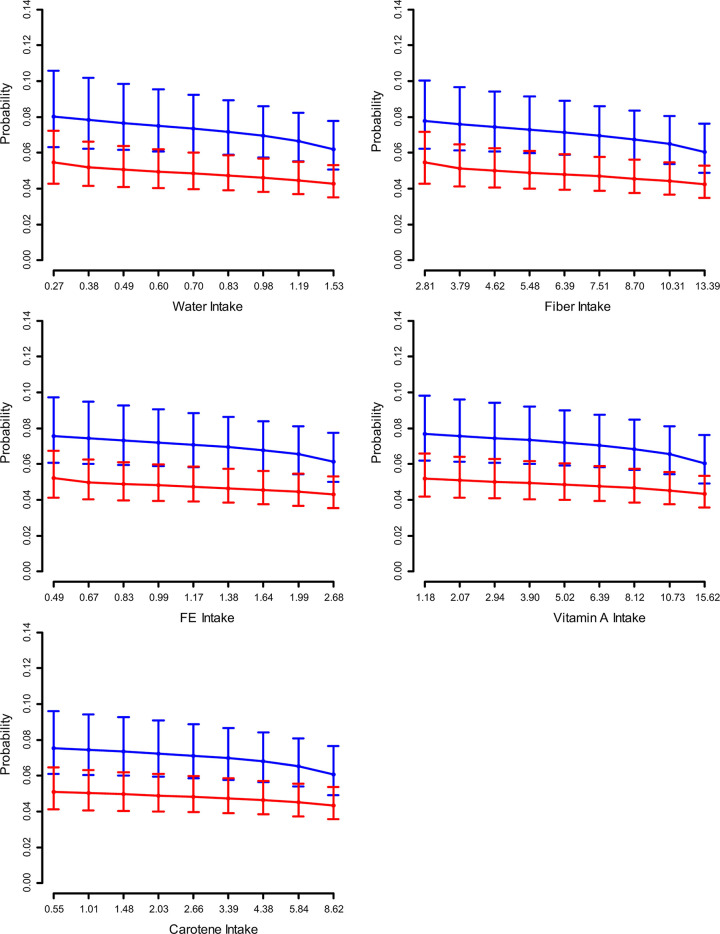
The incidence of ILD according to intake of water and some nutrients by participants. Participants were divided into 10 groups according to their dietary intake. The probability of having ILD decreased when intake of dietary factors (water, fiber, iron, vitamin A, and carotene) is increased. Two horizontal bars represent the 95% confidence interval.

When dietary intake was divided into quartiles among participants ≥50 years of age, the risk of having ILD was lower those with a higher intake of water, fiber, vitamin A, and carotene (Fig 1).

## Discussion

Our study revealed a significant correlation between dietary intake and laryngeal inflammation. A few previous studies have reported an association between dietary factors and laryngeal cancer [13, 22]. However, to our knowledge, ours is the first study to report an association between ILD and dietary factors using nationwide epidemiological data.

Comparison of participants with and without ILD, identified differences in the intake of coffee, fiber, and iron between the two group. Coffee intake of the ILD patients were higher than non-ILD participants with statistical significance. Coffee has previously been reported to lead to gastroesophageal reflux symptoms [23]. Caffeine is known to increase gastric reflux by lowering low esophageal sphincter tone. In laryngeal disease, increased coffee intake has been associated an increased risk of laryngeal cancer, resulting from recurrent mucosal injury by reflux material [24].

ILD patients tended to intake less iron and fiber than non-ILD participants. This association was also demonstrated in logistic regression analysis. Increased fiber and iron intake were significantly associated with decreased risk of having ILD. On subgroup analysis, by sex and age, the association between fiber and iron intake and ILD remained significant only for female and participants ≥50 years of age.

High dietary fiber intake is essential for maintaining general health and helps prevent colon cancer, diabetes, and cardiovascular disease [25, 26]. Dietary fiber affects regulatory T-cell numbers and functions through its effect on the gut microbiota and also has an effect on allergies and immune reactions of the respiratory tract [27, 28]. These immunomodulatory functions of dietary fiber could reduce the inflammatory reaction of the larynx. In laryngeal disease, in particular, an association between dietary fiber and laryngeal cancer has been reported [22]. Kawakita et al reported that dietary fiber decreased the risk of laryngeal cancer. Interestingly, this association was observed among patients ≥55 years old, but not in those <55 years old [22]. A fiber-enriched diet has also been reported to decrease gastric reflux by increasing esophageal sphincter pressure [29]. Since laryngeal reflux is a known etiological factor of chronic laryngitis, increased dietary fiber consumption could lower the risk for ILD.

The association between iron intake and laryngitis has not been reported previously. In our study, increased iron intake associated with decreased risk of having ILD. We do note a previous study reports of an association between a decreased iron intake among patients with non-erosive esophageal reflux disease [30]. Reflux from gastric lesion is major etiology of laryngeal inflammation; therefore, reflux might mediate the association between iron intake and ILD. Iron also has an effect on the immune function and gut microbiota which could further influence the association between iron intake and ILD. Since iron is essential for biological processes, an iron deficiency decreases human body function. On the other hand, an excess in iron has toxic effect on cells and increases the risk of gut bacterial infection. For these reasons, homeostasis of iron in the body is important. Our results demonstrated that iron intake is associated with ILD in female. Iron intake in our study was associated with ILD only among women, which might reflect the generally lower levels of iron in women than men.

Among individuals ≥50 years of age, increased vitamin A intake was associated with a lower risk of having ILD. Beneficial effects of vitamin A on cancer, chronic inflammatory disease, and vascular disease have previously been reported [15, 31–33]. With regard to laryngeal disease, vitamin A has been reported to have an effect on human papillomavirus infection and chronic laryngitis [19, 32]. Our results only revealed an association between vitamin A and ILD in the ≥50 age group. Vitamin A is particularly beneficial for patients who undergo radiation therapy or smoke owing to the antioxidant properties of vitamin A in scavenging reactive oxygen species [34]. As oxidative stress damage accumulates with aging, high consumption of vitamin A has been found to lower the risk for aging-related disease [35]. For this reason, sufficient vitamin A consumption would be important for individuals ≥50 years of age to lower the risk for ILD.

Our study has several strengths. This is the first large nationwide epidemiological study to investigate the association between dietary intake and ILD. Association between dietary factors and laryngeal cancer has been reported in several studies; however, to our knowledge, this is the first report of an association between micronutrients and laryngeal inflammation. Moreover, the KNHANES database collected from 2008 to 2012 is the only large and reliable nationwide database including direct observation of the larynx (using laryngoscope) and laryngeal disease diagnosed by laryngologists for all participants. The findings of our study could inform the development of specific dietary modification guidelines for ILD patients in different age and sex groups, considering the association with specific beverages and nutrients.

This study also has several limitations. First, as this is an observational, cross-sectional study, it is difficult to infer causality between dietary factors and ILD. ILD was defined as chronic laryngitis and contact granuloma. However, among the other diagnostic criteria, hyperkeratosis and even cancer have an inflammatory component. We excluded these two diseases due to their malignant or pre-malignant nature, to control for other dietary factors which might be more closely related to tumorigenesis than ILD. Dietary intake data in database is based on self-reporting, therefore, could be influenced by recall bias. Estimating nutrients using the 24-h dietary recall data is reported to be reliable, being facilitated by trained interviewers. However, because KNHANES data use a single 24-h dietary recall, the reliability of the average personal pattern of dietary intake may not be fully reflective of an individual’s dietary habits and, importantly, the intake of micronutrients, such as vitamin A, might be different from the true accumulated value over times. To overcome these limitations and to reveal temporal correlation between the nutrient intake and ILD, randomized controlled prospective clinical trials and animal-based basic research would be warranted.

## Conclusion

This cross-sectional nationwide study, using data from the fifth KNHANES in Korea, identified an association between dietary nutrient intake and ILD. Specifically, we identified that the risk for ILD was lowered by a decrease in coffee consumption and an increase in intake of iron and fiber. The association between ILD and nutrient intake was significant among individuals ≥50 years of age, with no association identified between dietary intake and ILD among individuals <50 years of age. Caffeine-containing drinks were associated with increased risk of having ILD, with differences by sex. Although the data type did not permit the identification of causality, our findings will be important to guide modifications in diet for patients with ILD, as well as for the prevention of ILD among individuals ≥50 years of age.
